# Initial development of the Sugar‐Sweetened Fruit Drink Questionnaire for examining beliefs, knowledge, and behaviors in an intervention to reduce sugar‐sweetened fruit drink intake in Alaska Native children

**DOI:** 10.1111/jphd.12639

**Published:** 2024-08-26

**Authors:** Todd C. Edwards, Cameron L. Randall, Courtney M. Hill, Scarlett Hopkins, Eliza Orr, Stephanie Cruz, Jeffrey Lee, Lloyd Mancl, Donald L. Chi

**Affiliations:** ^1^ Department of Health Systems and Population Health University of Washington Seattle Washington USA; ^2^ Department of Oral Health Sciences University of Washington Seattle Washington USA; ^3^ Department of Epidemiology University of Washington Seattle Washington USA; ^4^ Center for Alaska Native Health Research University of Alaska Fairbanks Fairbanks Alaska USA; ^5^ Oregon Health & Science University Portland Oregon USA

**Keywords:** Alaska Native communities, behavior, caregivers, child, oral health, sugar‐sweetened beverages

## Abstract

**Objective:**

Alaska Native children may be at increased risk for dental caries because of added sugar intake from sugar‐sweetened fruit drinks. This study describes development of a questionnaire to (a) assess Alaska Native caregivers' beliefs, knowledge, and behaviors regarding sugar‐sweetened fruit drinks, and (b) describe behavior changes within a community‐based intervention.

**Methods:**

Questionnaire development was conducted in three phases with Yup'ik Alaska Native caregivers in Southwest Alaska: (1) initial selection and adaptation of questionnaire items; (2) cognitive testing; and (3) data collection. The Sugar‐Sweetened Fruit Drink Questionnaire (SFDQ) contains 31 culturally‐tailored items across six areas: beliefs/values, environment/skills, knowledge, motivation, self‐efficacy, and behaviors.

**Results:**

Eighty‐one percent of caregivers gave their children sugar‐sweetened fruit drinks. Motivations included: what they grew up with (52%), few other options (46%), makes child happy (46%), healthier than soda (45%), and others in community drink them (42%). On average, 93% of caregivers believed drinking a lot of sugar‐sweetened fruit drinks leads to cavities in children and caregivers agreed (mean 4.1 on 5‐point scale, 5 = strongly agree) it is important to limit sugar‐sweetened fruit drinks. Among a sub‐sample of respondents (*n* = 20), we found low to moderate temporal stability in some SFDQ items over a 10–14 day period, indicating respondent ambivalence and/or uncertainty.

**Conclusions:**

Using community‐based participatory research methods, we developed a culturally tailored exploratory questionnaire that will be used to describe changes in caregiver knowledge, beliefs, attitudes, self‐efficacy, and behavior within a planned intervention to reduce sugar‐sweetened fruit drink intake in Alaska Native children.

## INTRODUCTION

American Indian and Alaska Native (AI/AN) children are disproportionately impacted by poor oral health, including dental caries [1]. For instance, AI/AN children aged 3–5 years have four times the prevalence of untreated dental caries than white children aged 3–5 years (43% vs. 11%) [1]. Attempts to address the dental caries epidemic in AI/AN communities have relied primarily on surgical approaches (e.g., fillings, crowns, extractions), which do not address health behaviors like poor diet and hygiene that are the root causes. At least one study reported that a motivational interviewing intervention failed to improve health behaviors or prevent dental caries in AI children [2]. An invited commentary on that study suggested that a potential explanation for the lack of improvement was that the intervention did not target specific behaviors linked to caries, like added sugar intake [3].

Sugar‐sweetened beverages (e.g., soft drinks, sugar‐sweetened fruit drinks, and energy drinks) are the most common source of added sugars among US children and adolescents [4] [5], and excess added sugar intake is a significant risk factor for dental caries in AI/AN communities [6]. Sugar‐sweetened fruit drinks like Tang® and Kool‐Aid® (T/K) are among the most commonly consumed foods and drinks in AN communities [5]. Historically, the traditional diet of Yup'ik AN people in Southwest Alaska was low in added sugars, but because of a nutrition transition in the past five decades, the Yup'ik AN diet has trended toward a Western diet, with less intake of traditional foods and increased intake of added sugars [7].

Many caregivers in the United States believe sugar‐sweetened fruit drinks are a healthy option for children [8]. Studies have found that children as young as 2 weeks of age are given sugar‐sweetened beverages, which is inconsistent with age‐based professional guidelines recommending no juice for children under age 12 months [9] [10]. Low caregiver health literacy contributes to this misperception [11]. This is a particular concern because caregivers are gatekeepers of beverages served at home, and caregivers' behaviors and child beverage intake are strongly associated [12].

The purpose of this work was to develop a questionnaire to collect information from caregivers that could be used to help develop and assess a community‐based sociobehavioral intervention aimed at reducing sugar‐sweetened fruit drink intake in Yup'ik AN children. We describe the process of developing an exploratory questionnaire to assess Yup'ik AN caregivers' knowledge, beliefs, and behaviors regarding sugar‐sweetened fruit drinks using community‐based participatory methods. The study contributes to the growing field of behavioral dentistry, in which such questionnaires can be used to study mechanisms underlying intervention effects, and underscores the importance of including community members in the questionnaire development process.

## METHODS

### Study design

There were three phases in the study: phase (1) initial selection and adaptation of 33 questionnaire items from the health behavior literature to craft the draft Sugar‐Sweetened Fruit Drink Questionnaire (SFDQ); phase (2) cognitive interview testing that resulted in a 44‐item draft SFDQ; and phase (3) data collection with the 44‐item draft SFDQ.

### Setting

This study was conducted with community members in the Yukon–Kuskokwim Delta (YK Delta) of Southwest Alaska. The YK Delta region is about the size of Oregon and is considered the homeland of Yup'ik and Cup'ik people, two related AN ethnic groups. More than 24,000 AN people live across 58 communities in the region and about 80% are Yup'ik ethnicity. Communities practice a mixed economy, whereby families engage in varying levels of a subsistence lifestyle (i.e., hunting, fishing, and gathering) and cash‐based employment. All communities are located off the road system; communities can only be reached by small plane year‐round, or by boat in the summer and snow machine in the winter.

### Ethics review

Written informed consent was obtained from all participants. The study was approved by the University of Washington Institutional Review Board, the Yukon‐Kuskokwim Health Corporation (YKHC) Human Studies Committee, and Tribal Councils in the study communities.

### Sampling

Study participants were enrolled through clinic‐based convenience sampling.

### Inclusion/exclusion criteria

Inclusion criteria were that the participant be 18 years of age or older, be able to communicate in English or Yup'ik, and, for phases 2 and 3, to have at least one child ages 10 years or younger (corresponding to the target age range for the intervention).

### Questionnaire development phases

#### Phase 1: Initial selection and adaptation of questionnaire items

Based on the health behavior literature and Bandura's Social Cognitive Theory, four areas were identified as determinants of sugar‐sweetened fruit drink intake in AN communities: beliefs, self‐efficacy, environment, and knowledge [8] [13] [14] [15]. The initial draft SFDQ consisted of 34 items adapted from past work [16, 17, 18] and included four SCT areas: beliefs/values (*n* = 8), environment/skills (*n* = 3), knowledge (*n* = 3), and self‐efficacy (*n* = 2). Additional items measuring motivation (*n* = 18) were included to assess the specific reasons why Yup'ik caregivers give their children sugared fruit drinks (and sugar‐free fruit drinks). Knowledge items had true or false response options. The remaining items had Likert‐type response scales with five categories: strongly agree, agree, no opinion/don't know, disagree, and strongly disagree. All items were written at the fifth grade level based on the Flesch–Kincaid reading level to ensure readability. All items were reviewed and modified as needed by a member of the research team who is a native Yup'ik speaker to ensure readability and cultural appropriateness. At every study stage, questionnaire items were translated into Yup'ik by the native speaker working with the study team.

#### Phase 2: cognitive testing

To assess clarity and comprehensibility of the draft questionnaire, we conducted in‐person cognitive interviews with 10 Yup'ik caregivers recruited from the YKHC dental clinic in Bethel. This sample size is consistent with established guidelines [18]. Caregivers had the option for the cognitive interview to be conducted in English or Yup'ik. Upon completing the 33‐item questionnaire, caregivers were asked questions aimed at assessing the cognitive attributes of each item. Standard procedures developed at the U.S. National Center for Health Statistics Cognitive Survey Laboratory were used in the debriefing process [19] with an emphasis on: (1) the degree to which participants found the item easy or difficult to complete; (2) whether participants had difficulties understanding item wording or interpreting response options; (3) cultural appropriateness and sensitivity of items; and (4) overall reaction to the questionnaire. Detailed notes were taken and compiled for decision‐making about questionnaire revisions. Each participating caregiver completed one cognitive interview. Based on findings from the cognitive interviews, 11 items were added to the questionnaire.

#### Phase 3: SFDQ data collection procedure

The SFDQ we collected data with included 44 items distributed across the areas: beliefs/values (8 items), environment/skills (8 items), knowledge (4 items), motivation (20 items), and self‐efficacy (2 items). Additional items were included under behavior (2 items) as direct measures of whether the caregiver gave their child sugared and sugar‐free fruit drinks. “Tang” and “Kool‐Aid” were used as examples of sugar‐sweetened fruit drinks, and “Crystal Light” (sugar‐free), “Sugar‐Free Tang” and “Sugar‐Free Kool‐Aid” were used as examples of sugar‐free drinks. These drink brands were chosen based on their local availability and intended target drinks in the intervention. In order to avoid response bias, we included a combination of positively, negatively, and neutrally worded questionnaire items referring to both sugar‐sweetened and sugar‐free drinks.

We collected SFDQ data from participants recruited from the same YKHC dental clinic in Bethel. The SFDQ was available in English or Yup'ik but was administered to all participants in English based on participant preference. A study staff member administered the SFDQ verbally to each participant in person to ensure consistency with the way questionnaire data would be collected during the intervention. Each participant was given a printed copy of the Likert‐type response scale, but not the items, to reference during administration of the SFDQ. Participant responses were recorded by the interviewer and manually entered into an Excel spreadsheet and verified for accuracy. Open‐ended responses and any participant comments were recorded.

Because this work was conducted in the context of developing a health promotion intervention, we included a follow‐up procedure to examine how fluid or stable participants' reported knowledge, beliefs, attitudes, self‐efficacy, and behaviors were regarding sugar‐sweetened beverages and child oral health promotion. Twenty percent of the total sample was randomly chosen for follow‐up. Participant ID numbers were selected a priori using a random number generator. Each follow‐up participant was contacted by telephone between 10 and 14 days of their baseline questionnaire and was re‐administered the SFDQ by telephone. All responses were recorded by the researcher.

### Participant recruitment

Phase 1 did not involve human study participants. For phases 2 and 3, we recruited Yup'ik caregivers through the YKHC dental clinic in Bethel, Alaska (the largest city in the YK Delta, with a population of about 6500). After check‐in, adults accompanying their child or visiting the clinic for their own dental appointment were approached in the waiting area by researchers who introduced the study and confirmed eligibility. For the cognitive interviews, each potential participant was informed that the interview would last approximately 30 min and was given a $25 gift card as a thank you for participating. For the SFDQ data collection, each potential participant was informed that the SFDQ administration would take 10–20 min, for which they would receive a $10 gift card as a thank you. The potential participants were also informed that 20% of the total sample would receive an invitation for repeat SFDQ administration by telephone within 2 weeks of the baseline administration, and that participation in the follow‐up would result in an additional $25 gift card.

### Analysis of SFDQ data

We computed standard descriptive statistics on the questionnaire data, examining frequency and patterns of missing data. For continuous measures, we computed means, medians, standard deviations (SD), and ranges. For categorical measures, we computed frequencies and modes. We checked for floor and ceiling effects using a threshold of 80% of responses in the bottom or top response categories. The threshold for missingness was missing responses at the item level for greater than 5% of the sample. We examined stability of the SFDQ individual items using Spearman r for ordinal scales and percent agreement for dichotomous response scales. To create the final SFDQ, we modified the preliminary questionnaire based on the results and improved readability, clarity, and utility in measuring aspects of the proposed behavioral intervention. We conducted statistical analyses with IBM SPSS statistics for windows, version 25.0 [20].

## RESULTS

### Study participants

We conducted cognitive interviews in January 2018, and the SFDQ data collection took place in April 2018 and October 2018. 10 participants were recruited for the cognitive interviews, and 106 participants were recruited for the SFDQ data collection, with 20 participants completing the SFDQ at baseline and follow‐up. In the SFDQ data collection 71% of participants were female, with a mean age of 35.6 years, and a mean number of 3.2 children living in the household. Participant characteristics for each phase of the study are presented in Table 1.

**TABLE 1:** Characteristics of study participants who completed cognitive interviews, sugar‐sweetened fruit drink questionnaire data collection and follow‐up in the Yukon‐Delta Kuskokwim region of Southwest Alaska.

	Cognitive interview *N* = 10	SFDQ data collection *N* = 106	SFDQ follow‐up *N* = 20
Sex, *n* (%)			
Male	1 (10%)	30 (28%)	5 (25%)
Female	9 (90%)	76 (71%)	15 (75%)
Preferred Language, *n* (%)			
English	8 (80%)	60 (56%)	11 (55%)
Yup'ik	0 (0%)	8 (7%)	1 (5%)
Both	2 (20%)	38 (35%)	8 (40%)
Yup'ik, *n* (%)			
Yes	10 (100%)	96 (90.6%)	18 (90%)
No	0 (0%)	9 (8.5%)	2 (10%)
Other	0 (0%)	1 (0.9%)	0 (0%)
Age (years), mean (SD)	31.6 (8.25)	35.6 (9.51)	34.4 (7.37)
Number of children in the household, mean (SD)	3.7 (1.57)	3.2 (1.82)	3.1 (1.62)

Abbreviations: CPG, Community Planning Groups; SD, standard deviation; SFDQ, Sugar‐Sweetened Fruit Drink Questionnaire.

### Cognitive interviews

Based on the cognitive interview results, we revised the draft SFDQ instructions and the wording and organization of the original 33 items. We simplified language where possible, added 3 items related to self‐efficacy, deleted 14 items that were considered redundant, and added 22 items to capture two additional areas: caregiver behaviors and motivations for giving children sugar‐sweetened or sugar‐free drinks. The behavior and motivations items were scaled as yes/no. This resulted in a net total of 11 items being added to the original 33‐item SDFQ (44 items total).

### SFDQ data collection

No SFDQ items exhibited floor or ceiling effects, and none had more than 5% of responses missing. The mean number of days between baseline and follow‐up SFDQ administration was 12 (range: 10–14 days). For the Likert scale items (beliefs/values, environment/skills, self‐efficacy), Spearman r between baseline and follow‐up ranged from −0.08 to 0.83 **(**Table 2 **)**. For the items with a yes/no response scale (knowledge, motivation, and behavior), percent agreement ranged from 55% to 95% **(**Table 3 **)**.

**TABLE 2:** Sugar‐Sweetened Fruit Drink Questionnaire item means and standard deviations at baseline (*n* = 106) and stability time 1–time 2 (*n* = 20).

Item stem (response scale: strongly agree = 5; agree = 4; no opinion = 3; disagree = 2; strongly disagree = 1)	Area	Mean	SD	Spearman r (T1–T2)
(1) **Tang® and Kool‐Aid® (T/K) healthy**	beliefs/values	2.27	1.00	0.83**
(2) **Important to limit T/K**	beliefs/values	4.11	0.79	0.61**
(3) Watering down T/K makes healthier	beliefs/values	2.82	1.02	0.17
(4) **Sugar‐free T/K healthier than regular**	beliefs/values	3.33	1.04	0.39
(5) **Sugar‐free T/K not as tasty as regular**	beliefs/values	3.28	0.75	0.27
(6) **Sugar‐free T/K are unsafe**	beliefs/values	2.67	0.80	0.26
(7) **Preventing cavities is important**	beliefs/values	4.60	0.49	0.24
(8) **Important to help child be cavity‐free**	beliefs/values	4.61	0.51	0.38
(9) Know how get child drink less sugared T/K	environment/skills	4.04	0.85	0.36
(10) Know how to tell from label difference between regular and sugar‐free drinks	environment/skills	4.20	0.64	0.46*
(11) **Sugar‐free T/K available in village**	environment/skills	3.45	1.13	0.39
(12**) Crystal Light available in village**	environment/skills	4.09	0.72	0.46*
(13) Know how get child try new drinks	environment/skills	3.91	0.77	0.41
(14) **Other adults give my child regular T/K**	environment/skills	3.36	1.08	0.76**
(15) Other adults support me encourage child drink sugar‐free T/K	environment/skills	3.58	1.08	0.17
(16) Know how get other adults give child less regular T/K	environment/skills	3.82	0.82	0.14
(17) **Can get child to drink less regular T/K**	self‐efficacy	4.07	0.72	−0.08
(18) **Can get child to drink sugar‐free T/K**	self‐efficacy	3.77	0.88	0.47*

*Note*: Bold represents item retained after analysis.

Abbreviation: SD, standard deviation.

* *p* < 0.05.

** *p* < 0.01.

**TABLE 3:** Sugar‐Sweetened Fruit Drink Questionnaire items percent endorsed yes at baseline (*n* = 106) and stability time 1–time 2 (*n* = 20).

Item stem (response alternatives: true or false)	Area	% yes	% agreement (T1–T2)
(19) **Tang® and Kool‐Aid® (T/K) contain vitamin C**	Knowledge	69	75
(20) **T/K healthier than soda**	Knowledge	52	85
(21) Drinking a lot of T/K leads to cavities	Knowledge	93	90
(22) **Sugar‐free T/K lead to cavities**	Knowledge	44	65
(23) **Give child sugared fruit drinks**	Behavior	81	75
Give child sugared fruit drinks because…			
(24) **Good replacement for fruits**	Motivation	16	80
(25) **Good treat for child**	Motivation	21	85
(26) **Inexpensive**	Motivation	31	90
(27) Calm down/soothe child	Motivation	22	90
(28) **Make child happy**	Motivation	46	80
(29) **Others in my village drink them**	Motivation	42	80
(30) **Healthier than soda**	Motivation	45	55
(31) Our water tastes or looks bad	Motivation	14	90
(32) **There are few other drink options**	Motivation	46	70
(33) **They are what I grew up with**	Motivation	52	80
(34) **Give child sugar‐free fruit drinks**	Behavior	62	95
Give child sugar‐free fruit drinks because…			
(35) **Good replacement for fruits**	Motivation	20	65
(36) **Good treat for child**	Motivation	25	80
(37) **Inexpensive**	Motivation	20	90
(38) Calm down/soothe child	Motivation	22	70
(39) **Make child happy**	Motivation	34	75
(40) **Others in my village drink them**	Motivation	18	90
(41) **Healthier than soda**	Motivation	50	75
(42) Our water tastes or looks bad	Motivation	11	85
(43) There are few other drink options	Motivation	25	75
(44) They are what I grew up with	Motivation	21	90

*Note*: Bold represents item retained after analysis.

### SFDQ responses

#### Beliefs and values

Caregivers modestly disagreed that sugar‐sweetened T/K are healthy for their children (*M* = 2.27, SD = 1.00) and agreed that it is important to limit how much T/K their children drink (*M* = 4.11, SD = 0.79). They strongly agreed that preventing cavities in their children is important (*M* = 4.60, SD = 0.49) and that it is important to help their children be cavity‐free (*M* = 4.61, SD = 0.51). The Spearman correlations for the latter two items were nonsignificant however, indicating potential ambivalence or uncertainty (0.24 and 0.38, respectively). Caregivers were somewhat unsure if sugar‐free T/K are unsafe (*M* = 2.67, SD = 0.80) and were unsure about whether sugar‐free T/K are healthier than T/K with sugar (*M* = 3.33, SD = 1.04). The Spearman correlations of these items were also nonsignificant (Table 2).

#### Environment and skills

Caregivers agreed that they knew how to get their children to drink less sugar‐sweetened T/K (*M* = 4.04, SD = 0.85). They were unsure whether sugar‐free T/K was available in their village (*M* = 3.45, SD = 1.13), but agreed that Crystal Light is available (*M* = 4.09, SD = 0.72). The Spearman correlations for these items were nonsignificant, however. Caregivers were unsure whether other adults in their home gave their children sugar‐sweetened T/K (*M* = 3.36, SD = 1.08). They agreed that they knew how to tell the difference between sugar‐sweetened and sugar‐free drinks based on the product label (*M* = 4.20, SD = 0.64) (Table 2).

#### Knowledge

69% responded (75% agreement) of caregivers responded yes that sugar‐sweetened T/K contain vitamin C, 52% that sugar‐sweetened T/K are healthier than soda (85% agreement), and 93% that drinking a lot of T/K leads to cavities (90% agreement) (Table 3).

#### Motivations

Caregivers' most common motivations for giving their children sugar‐sweetened fruit drinks were because: they are what they grew up with (52%, with 80% agreement), there are few other options (46%, with 70% agreement), they make their children happy (46%, with 80% agreement), they are healthier than soda (45%, with 55% agreement), and others in their village drink them (42%, with 80% agreement). The most common motivation reported for giving sugar‐free fruit drinks was that they are healthier than soda (50%, with 75% agreement) (Table 3).

#### Self‐efficacy

Caregivers agreed that they would be able to get their children to drink less sugar‐sweetened T/K (*M* = 4.07, SD = 0.72), but were less sure if they could get their children to drink sugar‐free T/K (*M* = 3.77, SD = 0.88). The Spearman correlation for ability to get child to drink fewer sugar‐sweetened drinks was −0.08 however, indicating strong uncertainty (Table 2).

#### Behaviors

Around 81% (75% agreement) of caregivers reported giving their children sugar‐sweetened fruit drinks, and 62% (95% agreement) gave their children sugar‐free drinks (Table 3).

### Final SFDQ

We dropped 13 items after reviewing the initial results. Examples of items dropped included: “I know how to get my child to try new drinks” and “I give my child sugar‐sweetened fruit drinks because our water tastes or looks bad.” The first item was considered to be too general and the second was not considered to be essential for inclusion and was potentially stigmatizing of local water sources. Thirty‐one items were retained in the final instrument: beliefs/values (7 items), environment/skills (3 items), knowledge (3 items), motivation (14 items), self‐efficacy (2 items), and behavior (2 items). The final SFDQ items are bolded in Tables 2 and 3.

## DISCUSSION

In this study, we developed a questionnaire for future use in behavioral interventions to address sugar‐sweetened fruit drink intake in Yup'ik AN children. The final 31‐item questionnaire went through multiple stages of revisions and is ready to be used in the intervention to collect information regarding caregivers' beliefs and values, environment and skills, knowledge, motivation, self‐efficacy, and behaviors regarding intake of sugar‐sweetened and sugar‐free fruit drinks. We found widespread reporting that Yup'ik caregivers gave their children sugar‐sweetened fruit drinks, which is consistent with previous research indicating that sugar‐sweetened fruit drinks are the most frequently consumed foods or drinks in AN communities [5]. We also found that caregivers were unsure about whether sugar‐free T/K were healthier than those with sugar, and one‐half of the participants believed that sugar‐sweetened T/K were healthier than soda, mirroring research in the general United States that reports many caregivers believe some sugary drinks are healthy options for children [8]. While caregivers on average strongly agreed that preventing cavities is important, we found there was uncertainty around this belief. We also found that caregivers seemed uncertain about whether they could get their children to drink less sugar‐sweetened T/K. These are targets that were incorporated into our intervention.

Two main limitations should be considered in the interpretation of our study. First, although the results described in the study went through multiple stages of community stakeholder review, due to sample size restrictions these results should be considered preliminary. Second, it would have been ideal to conduct activities in the actual communities that would be part of the intervention rather than in Bethel, but the communities are small and doing so would have potentially confounded intervention results. However, parents in Bethel are similar to individuals in the study communities given that most families routinely travel from outlying communities to Bethel for health care services.

This work advances the field of oral health research in multiple ways. First, we developed the SFDQ using community‐based participatory research methods, which is the recommended practice for conducting research with Indigenous communities. Community‐based participatory research aims to alleviate health disparities in a way that recognizes and overturns historical power differentials [21] [22]. Second, we aimed to intervene on a behavioral determinant of oral health disparities in remote AN communities, which improves on solely surgical approaches to treat dental diseases. Third, this study applies behavioral and social science methods to oral health promotion and highlights the crucial role that racial, cultural, and other socioeconomic contrasts have on the burden of oral health diseases.

In terms of relevance for future interventions, sugar‐free water enhancers are a promising potential replacement option for sugar‐sweetened beverages. These commercially available water flavoring drops come in the same flavors as popular sugar‐sweetened fruit drinks like T/K. The water enhancers contain artificial sweeteners (e.g., sucralose and acesulfame‐potassium), are safe in the small quantities consumed by children [23] [24], and are acceptable to children [25]. Even small reductions in added sugar intake can prevent obesity and diabetes, and reduce blood pressure, triglycerides, and cholesterol [26] [27] [28] [13]. In vitro research also shows that sugar‐free Tang® is not cariogenic [26] [29]. However, local stores in many underserved communities, including those in AN communities, do not stock sugar‐free water enhancers because they are relatively new to the market and have no consumer demand. Many parents also may be reluctant to introduce their children to new beverages and lack self‐efficacy skills to effectively anticipate and address resistance, which are barriers to sustained behavior change [27] [28]. A community‐based intervention that was designed to account for these multiple barriers to switching to sugar‐free beverages is currently being evaluated (see https://reporter.nih.gov/project-details/10192501).

In conclusion, added sugar intake from sugar‐sweetened fruit drinks contributes to the caries epidemic among AN children. In response, we developed a questionnaire to assess AN Yup'ik caregivers' knowledge, beliefs, and behaviors regarding sugar‐sweetened fruit drinks using community‐based participatory methods. The SFDQ will be used to examine knowledge, beliefs and behaviors within a community‐based intervention. Findings of the questionnaire development study showed that intake of sugar‐sweetened fruit drinks is prevalent and that there may be some caregiver indecision around beliefs and motivations. This work advances the field of behavioral dentistry by underscoring the importance of including community members in the questionnaire development process and by developing behavioral‐based interventions to address oral health disparities.

## FUNDING INFORMATION

NIH/NIDCR Grant Nos. R56DE025813, U01DE027629, and R01DE026741‐02S1.

## CONFLICT OF INTEREST STATEMENT

The authors have no conflicts of interest to disclose.

## PATIENT CONSENT STATEMENT

All study participants provided informed consent.

## Data Availability

The data that support the findings of this study are available from the corresponding author upon reasonable request.
